# Dynamic Cross-Modal Modeling with an Ultra-Lightweight Architecture for Face Anti-Spoofing

**DOI:** 10.3390/s26092699

**Published:** 2026-04-27

**Authors:** Nana Li, Jiayu Wang, Zuhe Li, Zhipeng Weng, Yihong Wang, Yushan Pan

**Affiliations:** 1 School of Computer Science and Artificial Intelligence, Zhengzhou University of Light Industry, Zhengzhou 450002, China; linana@zzuli.edu.cn (N.L.); zuheli@zzuli.edu.cn (Z.L.); 332305060494@zzuli.edu.cn (Z.W.); 2 Department of Computing, Xi’an Jiaotong-Liverpool University, Suzhou 215123, China; yihong.wang@liverpool.ac.uk (Y.W.); yushan.pan@ieee.org (Y.P.)

**Keywords:** face anti-spoofing, dynamic cross-modal modeling, multimodal fusion, lightweight architecture

## Abstract

The effectiveness of multimodal face anti-spoofing largely depends on the modeling of cross-modal relationships. However, most existing approaches rely on static fusion or implicitly learned feature aggregation, which assumes fixed modality importance, limiting its ability to capture reliability variations across different attack patterns. Under strict computational constraints, achieving effective dynamic cross-modal modeling remains a significant challenge. To address this issue, we propose an ultra-lightweight dynamic cross-modal framework for face anti-spoofing, with ultra-low parameters, FLOPs, latency, memory and high FPS for real-time edge inference. A compact feature extractor is constructed by enhancing ShuffleNetV2 with the Ghost-Generated Shuffle BlockA (GGS-BlockA), which significantly reduces redundant computation while maintaining high discriminative capability. On this basis, a Lightweight Cross-Modal Attention (LCMA) module performs sample-wise dynamic modality reweighting to capture reliability variations among RGB, Depth, and IR modalities. Furthermore, a Lightweight Cross-Modal Fusion (LCMF) module utilizes depth cues as stable guidance to improve cross-modal feature alignment and complementary representation. Experiments on the CASIA-SURF benchmark demonstrate that the proposed method achieves an Average Classification Error Rate (ACER) of 0.064% with only 0.14M parameters and 0.0065G FLOPs. At the strict threshold of $\mathrm{TPR}@\mathrm{FPR}=10^{-4}$, a detection rate of 99.86% is obtained, demonstrating strong robustness and generalization capability under extremely low computational cost.

## 1. Introduction

Driven by recent advances in artificial intelligence and computer vision, face recognition technology has been widely applied in identity verification, mobile payment, and public security systems. However, as deployment scale continues to grow, security threats from presentation attacks have become increasingly prominent. Adversaries use printed photos, video playback, 3D masks, and other spoofing methods to deceive recognition systems, severely threatening reliability and security. Face anti-spoofing has therefore evolved from an auxiliary component into a core module for secure face recognition. As attack types grow more diverse and realistic, accurately distinguishing bona fide faces from sophisticated spoofs remains a critical challenge that demands further investigation.

Early face anti-spoofing methods mainly focus on the RGB modality, using handcrafted features or deep appearance patterns based on texture, frequency, or temporal clues [1]. Although effective in controlled environments, such approaches rely heavily on 2D appearance cues and suffer severe performance degradation under illumination changes, sensor variations, or high-fidelity attacks, limiting real-world generalization [2]. To alleviate these issues, recent studies have introduced auxiliary modalities such as depth and infrared, exploiting complementary information via multimodal fusion [3]. Extensive benchmarks confirm that multimodal methods consistently surpass unimodal approaches in both accuracy and stability [4].

Nevertheless, such gains often incur considerable computational overhead. Furthermore, existing works typically address fusion quality and efficiency in isolation. Simply lightening existing dynamic fusion models tends to weaken fine-grained cross-modal modeling, as these methods typically rely on multi-branch structures or high-dimensional attention mechanisms to capture complex cross-modal interactions. Conversely, directly injecting dynamic mechanisms into lightweight architectures often introduces non-negligible extra cost. This is because even relatively lightweight attention operations can offset the efficiency advantages of compact backbone networks. Thus, simultaneously achieving adaptive cross-modal modeling and strict computational efficiency remains highly challenging. This indicates an inherent trade-off between modeling capability and efficiency, which cannot be effectively resolved through straightforward adaptations of existing methods. Complex multi-branch structures and high-dimensional fusion operations increase model size and latency, hindering deployment on resource-constrained edge devices [5].

More importantly, modality contributions are not fixed but vary across spatial regions, attack types, and capture conditions. While depth often provides reliable structural cues and may dominate in some scenarios, its effectiveness degrades under sensor noise, low-quality capture, or sophisticated 3D attacks. In contrast, RGB and infrared supply complementary texture and reflectance information, indicating that modality importance is input-dependent rather than consistently dominated by a single source. Most existing fusion strategies rely on static or coarse-grained rules that cannot fully capture such variations, leading to vulnerability to modality noise, missing inputs, and unseen attacks [6]. Therefore, a lightweight yet dynamically adaptive fusion framework is urgently needed to integrate multimodal cues effectively and enable robust, efficient anti-spoofing in real-world edge scenarios.

Motivated by the above observations, this work explores efficient and adaptive cross-modal fusion under tight computational constraints, targeting robust face anti-spoofing on practical edge platforms. To address these challenges jointly, we employ a co-design strategy that unifies lightweight architecture and dynamic cross-modal interaction within a unified framework. We propose LAM-FAS, an ultra-lightweight framework for dynamic cross-modal modeling in face anti-spoofing.

Built on a co-design philosophy that unifies efficient architecture and adaptive cross-modal modeling, LAM-FAS dynamically estimates sample-aware modality importance instead of using fixed fusion weights. By coupling low-cost feature extraction and input-dependent cross-modal attention from the ground up, the framework achieves strong robustness against diverse attacks while maintaining extreme efficiency for real-time edge deployment. The main contributions of this work are summarized as follows:

(1) We propose an ultra-lightweight architecture for dynamic cross-modal modeling in multimodal face anti-spoofing. Unlike conventional methods that simply append fusion modules to lightweight networks, the proposed framework jointly considers structural compression and cross-modal interaction during architectural design. A compact feature extractor is constructed by enhancing ShuffleNetV2 with the proposed GGS-BlockA module, significantly reducing redundant computation while preserving discriminative representation capability.
(2) We design a Lightweight Cross-Modal Attention module (LCMA) to explicitly model input-dependent relationships among RGB, Depth, and IR modalities. The module performs sample-wise dynamic modality reweighting to capture reliability variations across attack patterns, enabling adaptive cross-modal interaction rather than fixed modality contributions.
(3) We develop a Lightweight Cross-Modal Fusion module (LCMF) guided by depth-aware weight learning for robust multimodal feature integration. By using depth cues as stable guidance, the module promotes consistent cross-modal alignment and strengthens complementary representations, further improving robustness and generalization under complex and unseen attacks.

## 2. Related Work

### 2.1. Face Anti-Spoofing with Single- and Multi-Modal Inputs

Early research on face anti-spoofing (FAS) primarily focused on RGB imagery, as it constitutes the standard output format of most commercial imaging devices. The main objective of these studies was to exploit appearance-based cues—such as texture, color distribution, and spatial structural patterns—to distinguish bona fide facial samples from common presentation attacks, including printed photographs and replayed screen content. With the advancement of deep learning techniques, the field gradually shifted from handcrafted feature engineering to data-driven representation learning using convolutional neural networks (CNNs). Early on, Boulkenafet et al. [7] showed the effectiveness of color–texture analysis under varying lighting conditions. Atoum et al. [8] added depth as a supervisory signal to help CNNs focus on local patches. Deb and Jain [9] combined local texture modeling with global reasoning to improve cross-dataset generalization. More recently, Yu et al. [10] explored physiological signals such as rPPG with Transformers to detect high-quality 3D mask attacks. While single-modal approaches are simple and efficient, they degrade significantly under lighting changes, sensor variations, or realistic spoofs [10]. This limitation has driven the development of multimodal FAS systems. By fusing complementary cues from RGB, depth, and infrared, multimodal methods achieve stronger robustness than unimodal counterparts [3]. Parkin and Grinchuk [11] showed that joint multimodal feature modeling improves spoof detection. The release of the CASIA-SURF dataset [3] and its attention-based fusion framework further accelerated progress in this area.

Later innovations include fine-grained local learning [12], multi-level feature fusion [13], and Transformer-based cross-modal dependency modeling [14]. Cascaded strategies that combine feature and decision fusion were also explored [15]. Recent studies further improved representation and generalization ability, including convolutional vision Transformer hybrids [16] and one-class FAS frameworks for unseen attacks [17]. SLIP [18] further enhanced generalization via vision–language pretraining. However, most multimodal FAS methods still rely on static or implicitly learned fusion strategies [11,13]. Typical static schemes include feature concatenation and score-level averaging, which assume fixed modality importance across samples. This assumption is unrealistic in practice. Modality reliability varies dynamically with attack types, environments, and sensor quality [3]. Recent studies also validate that such variation is critical for practical anti-spoofing [17]. For example, depth can degrade under reflective surfaces or high-quality 3D masks. Infrared signals may suffer from strong ambient light interference. Importantly, depth does not consistently dominate all attack scenarios [19], even if it performs well in single-modal tests. Static fusion thus cannot adapt to modality imbalance and may introduce misleading information [19]. To address these issues, recent dynamic fusion methods use attention or cross-modal interaction to realize input-dependent modality reweighting [20]. These approaches improve robustness against complex and unseen attacks [21]. However, they often rely on multi-branch structures or heavy attention modules, leading to high computation costs. Some efforts have been made toward lightweight mobile FAS systems [21] and generalized fusion [22]. Spatial and channel attention mechanisms have also been studied [23]. Adversarial cross-modality translation further improves cross-modal modeling [24]. Nevertheless, few methods achieve efficient dynamic cross-modal modeling under strict computational constraints. This is largely because dynamic fusion and lightweight design are often treated as independent problems, making their joint optimization challenging.

### 2.2. Lightweight Network Architectures for Face Anti-Spoofing

As the demand for real-time face anti-spoofing (FAS) grows, particularly for deployment on mobile and embedded devices, the challenge lies in developing models that are not only accurate but also computationally efficient. Traditional deep learning architectures, such as VGG and ResNet, deliver strong performance but are often too computationally and memory-intensive for resource-constrained environments. Consequently, the community has been actively developing lightweight designs that maintain accuracy while reducing computational cost. Much of this progress stems from improving convolution operations, including depthwise separable convolutions, channel shuffling, and low-cost feature generation. For instance, MobileNet [25,26] decomposes standard convolutions into depthwise and pointwise operations to reduce computation; ShuffleNet [27,28] enhances cross-channel communication through channel shuffling; and GhostNet [29] further reduces redundancy by generating “ghost” feature maps via computationally inexpensive linear operations. Owing to their favorable balance between efficiency and representation capability, these backbones have become popular foundations for practical FAS systems.

Building on these lightweight backbones, more specialized models have been developed to achieve better efficiency–accuracy trade-offs for FAS; for example, FeatherNet [5], derived from MobileNetV2, achieves high efficiency with approximately 0.35M parameters, while NAS-FAS [30] leverages neural architecture search to obtain compact task-specific designs with around 0.27M parameters. Beyond model compression, researchers have also focused on enhancing discriminative capability; for instance, the RSGB block [2] improves gradient-based artifact detection, while uncertainty modeling [31] and artifact disentanglement [32] address more challenging attack scenarios. In addition, lightweight attention modules such as SE [33] and CBAM [34] have been incorporated to adaptively re-weight features with minimal overhead. However, when extending these designs to multimodal FAS—incorporating depth, infrared, or other modalities—lightweight backbones alone are insufficient. The introduction of additional branches and fusion operations often offsets the efficiency gains. Recent studies have suggested that embedding attention mechanisms into lightweight multimodal architectures can partially alleviate this issue [35,36]. Related efforts on multimodal attention and cross-modal modeling further support this direction [23,24]. Nevertheless, most existing approaches treat backbone design and fusion strategy as largely independent components. As a result, structural efficiency and cross-modal interaction modeling are rarely jointly optimized, particularly when input-dependent modality variations must be handled under strict computational constraints. Moreover, while lightweight architectures effectively reduce computational cost, they are primarily designed for single-modal or independently processed features. When extended to multimodal settings, the additional complexity introduced by cross-modal fusion often diminishes their efficiency advantages. This observation highlights the necessity of jointly considering lightweight design and adaptive fusion mechanisms for dynamic cross-modal modeling in multimodal face anti-spoofing.

### 2.3. Cross-Modal Fusion and Attention Mechanisms for Face Anti-Spoofing

Cross-modal fusion plays a critical role in multimodal face anti-spoofing, as it determines how complementary information from RGB, depth, and infrared (IR) modalities is integrated. Existing fusion strategies can generally be categorized into static and dynamic approaches. Early methods mainly adopt static designs, such as feature concatenation, fixed-weight averaging, or predefined fusion rules [11,13,37]. While computationally efficient, these approaches assume consistent modality contributions across samples. However, in practice, modality reliability is highly input-dependent and varies with attack types, environmental conditions, and sensor noise [3,19]; for example, depth information may degrade under reflective or mask-based attacks, while infrared signals can be affected by strong ambient illumination. Importantly, no single modality—including depth—is consistently dominant across all scenarios [19], which limits the effectiveness of fixed fusion strategies. As a result, static methods often fail to handle modality imbalance and may introduce redundant or misleading information.

To address these limitations, recent studies have explored dynamic fusion mechanisms that explicitly model cross-modal interactions. Attention-based methods and Transformer architectures enable input-dependent modality reweighting. For instance, Conv-MLP [38] captures both local and global representations, while cascaded fusion strategies [15] progressively refine multi-level features. Cross-attention mechanisms [39] further enhance robustness by dynamically adjusting modality contributions under varying conditions. Although effective, these approaches typically rely on multi-branch structures, high-dimensional interactions, or computationally intensive attention modules, leading to increased model complexity and inference cost. Another practical challenge is the presence of missing or unreliable modalities due to sensor failure, occlusion, or transmission issues. To mitigate this, cross-modal feature transition mechanisms have been proposed [19], allowing models to compensate for missing modalities via learned inter-modal relationships. However, such methods often introduce additional complexity and are not optimized for lightweight deployment. In parallel, recent advances have explored more powerful representation learning strategies. Convolutional vision Transformer hybrids [16] improve feature modeling by combining local and global representations, while one-class face anti-spoofing methods [17,18] enhance generalization by learning discriminative spoof cues or leveraging vision-language pretraining. Nevertheless, these approaches primarily focus on generalization and do not explicitly address efficient cross-modal interaction under strict computational constraints. Overall, while dynamic fusion improves adaptability and robustness compared to static methods, existing approaches either incur high computational overhead or lack robustness to missing modality scenarios. Therefore, designing an efficient, lightweight, and adaptive cross-modal fusion framework remains a critical challenge in multimodal face anti-spoofing.

## 3. Proposed Method

### 3.1. Overall Framework of the Proposed LAM-FAS

**Figure 1:**
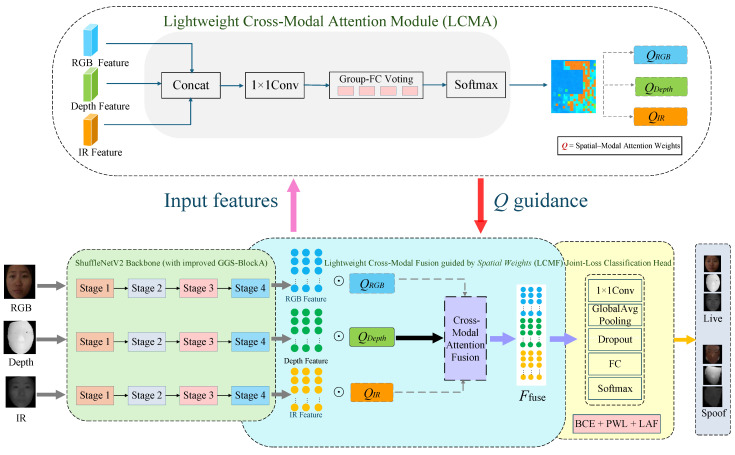
Overall architecture of the proposed LAM-FAS framework.

We propose LAM-FAS, an ultra-lightweight architecture for dynamic cross-modal modeling in face anti-spoofing, designed to explicitly capture input-dependent modality reliability under strict computational constraints. In this work, “ultra-lightweight” refers to a design with extremely low parameters, FLOPs, inference latency, and memory usage, while achieving high FPS for efficient real-time deployment on resource-constrained edge devices. The overall architecture is illustrated in Figure 1. LAM-FAS comprises three principal components: an enhanced ShuffleNetV2-based lightweight backbone for multimodal feature extraction, a Lightweight Cross-Modal Attention (LCMA) module, and a Lightweight Cross-Modal Fusion (LCMF) module. Given RGB, Depth, and Infrared (IR) inputs, the shared lightweight backbone extracts modality-specific feature representations, denoted as $\left\{F_{\mathrm{RGB}},F_{\mathrm{Depth}},F_{\mathrm{IR}}\right\}$. These features are subsequently fed into the LCMA module to generate an input-dependent spatial–modal weight map *Q*, which captures the relative reliability of each modality across spatial locations. Conditioned on *Q*, the LCMF module performs selective reweighting and efficient cross-modal aggregation to produce a compact and discriminative fused representation $F_{\mathrm{fusion}}$. In particular, depth information is leveraged as a relatively stable structural cue to guide the fusion process, facilitating more reliable cross-modal feature alignment and complementary interaction among RGB, Depth, and IR modalities. The resulting fused representation is then fed into a lightweight classification head for liveness prediction. The entire network is optimized end-to-end using a joint supervision objective.

### 3.2. Ghost-Generated Shuffle Block A (GGS-BlockA)

**Figure 2:**
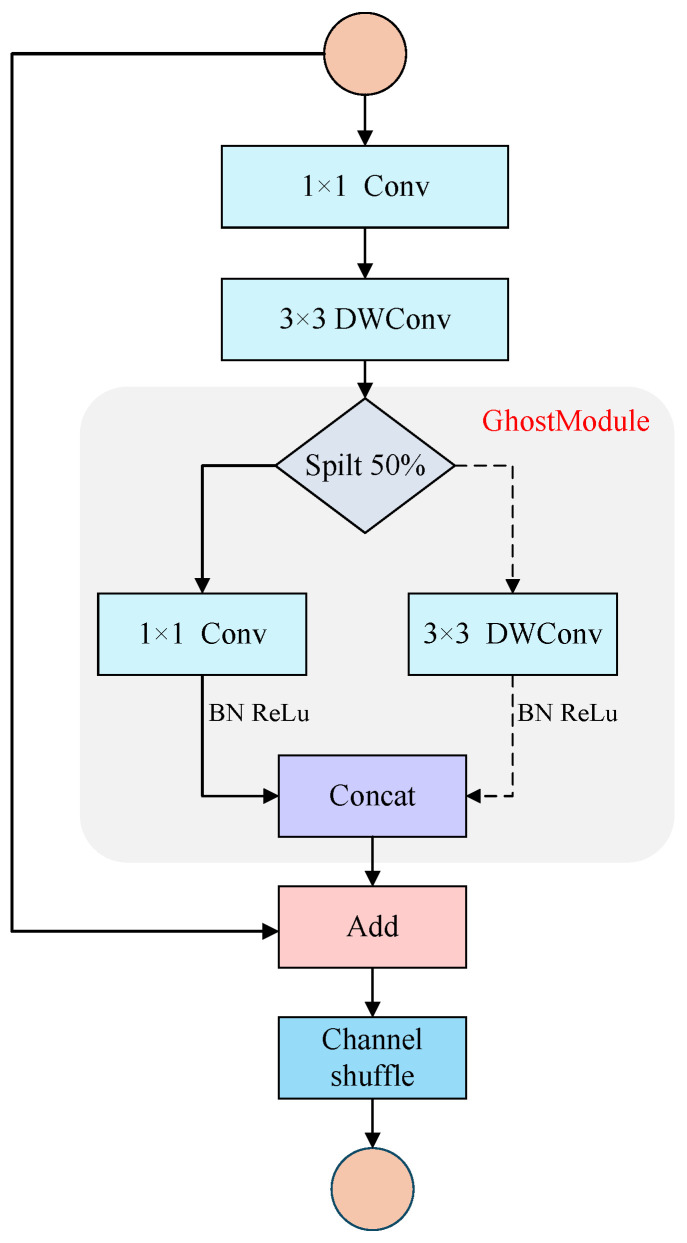
Architecture of the proposed lightweight GGS-BlockA module.

Following the lightweight design principles of the ShuffleNet family [27,28], ShuffleNetV2 is adopted as the backbone and its basic unit is refined to better balance efficiency and representational capacity for multimodal face anti-spoofing. Although ShuffleNetV2 achieves high efficiency through channel splitting and a branch-based architecture, its original block exhibits limited feature reuse and reduced flexibility when processing heterogeneous inputs such as RGB, depth, and infrared (IR) modalities. Compared with conventional lightweight designs that mainly rely on channel compression or depthwise separable convolutions, Ghost modules explicitly exploit feature redundancy to generate additional feature maps through cheap linear operations, thereby improving feature diversity without increasing computational burden, making them particularly suitable for maintaining representational capacity under strict computational constraints. To address this limitation, the feature generation strategy of GhostNet [29] is incorporated to construct a lightweight feature extractor termed the Ghost-Generated Shuffle Block A (GGS-BlockA). The proposed block preserves the overall structure of the ShuffleNetV2 unit while enhancing feature diversity through Ghost-style feature generation with negligible computational overhead. Specifically, the input feature first passes through a 1×1 convolution for channel adjustment, followed by a 3×3 depthwise convolution for efficient spatial modeling. Subsequently, a dual-branch Ghost module employs low-cost linear transformations to generate additional feature maps, enriching representation capacity without introducing significant computation. The generated features are concatenated and subjected to channel shuffling to facilitate inter-channel information exchange and improve feature utilization. When the stride equals 1 and the input and output channels are identical, a residual connection is introduced to stabilize gradient propagation and accelerate convergence, as illustrated in Figure 2.

**Figure 3:**
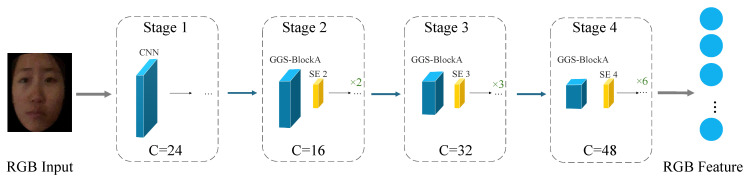
Feature extraction process of the modified ShuffleNetV2 backbone, illustrated using the RGB branch as an example. All modality branches share the same backbone architecture.

The structural design of GGS-BlockA jointly considers efficiency and performance. By employing 1×1 convolutions for channel reduction, a 2:1 branch partition strategy, and a cheap feature reuse mechanism, the proposed module maintains parameter count and computational cost (FLOPs) at the same order of magnitude as the original ShuffleNetV2 block, thereby satisfying the deployment requirements of resource-constrained devices. Meanwhile, through the synergistic integration of Ghost module principles, grouped convolutions, and channel shuffle operations, GGS-BlockA improves the utilization efficiency and discriminative capability of multi-modal features. The backbone network follows a lightweight design strategy with modality-wise independent feature extraction and progressive downsampling. At the input stage, the RGB, Depth, and IR branches are each processed by a unified 3×3 convolution to perform spatial downsampling and channel expansion to 24 channels, producing shallow feature maps of size [B,24,32,32]. Subsequently, the three modalities independently stack Stage2–Stage4 in parallel, where each stage is built upon GGS-BlockA as the basic unit. At the end of each stage, a Squeeze-and-Excitation (SE) channel attention module is embedded to enable lightweight intra-modal feature recalibration while suppressing background noise. The detailed configuration is as follows. Stage2 cascades 2× GGS-BlockA with SE2, reducing the spatial resolution to 16×16 with 16 output channels. Stage3 stacks 6× GGS-BlockA with SE3, further downsampling the feature maps to 8×8 and expanding the channel dimension to 32. Stage4 cascades 3× GGS-BlockA with SE4, finally producing high-level semantic feature maps of size 4×4×48, as illustrated in Figure 3. Under an extremely limited parameter budget, the entire backbone completes a three-level feature extraction process spanning shallow texture, mid-level structural patterns, and high-level semantic representations, thereby providing compact yet expressive modality-specific features that serve as an efficient foundation for subsequent dynamic cross-modal interaction modeling.

### 3.3. Lightweight Cross-Modal Attention Module (LCMA)

The LCMA module performs lightweight and adaptive cross-modal attention modeling for RGB, Depth, and IR features. In multimodal face anti-spoofing, different modalities often exhibit distinct reliability across spatial regions and attack types. Existing fusion strategies typically rely on implicit or globally shared weighting schemes, which fail to capture such input-dependent variations. To address this issue, LCMA explicitly models modality-aware attention at the pixel level, enabling dynamic adjustment of modality contributions.

Given the tri-modal feature maps $F_{m}\in R^{B\times C\times H\times W}$, where m∈{RGB,Depth,IR}, we first concatenate the features along the channel dimension to form a fused representation:

(1) $$F=\mathrm{Concat}\left(F_{\mathrm{RGB}},F_{\mathrm{Depth}},F_{\mathrm{IR}}\right)\in R^{B\times 3C\times H\times W}.$$

To improve efficiency and reduce redundancy, a 1×1 convolution is adopted to compress the channel dimension to C/8:

(2) $$\tilde{F}=\phi \left(F\right),\;\tilde{F}\in R^{B\times C/8\times H\times W},$$

where ϕ(·) denotes the channel compression operation. This design follows the common practice in lightweight attention mechanisms (e.g., SE [33]), striking a balance between representation capacity and computational cost.

Based on the compact features, LCMA employs grouped 1D convolutions to model cross-modal interactions along the channel dimension:

(3) $$R={\mathrm{Conv}1D}_{g}\left(\tilde{F}\right),$$

where the number of groups is set to g=4. Compared with standard 2D convolutions, 1D convolutions focus on capturing cross-modal dependencies while avoiding unnecessary spatial computation. The grouping strategy further reduces parameter overhead and enables efficient parallel processing over channel subsets, achieving a practical trade-off between expressiveness and efficiency.

The resulting responses are reshaped and aggregated, followed by Softmax normalization to generate pixel-wise modality weight maps:

(4) $$Q_{m}=\frac{\exp({\bar{R}}_{m})}{\sum_{m^{\prime }}\exp\left({\bar{R}}_{m^{\prime }}\right)},\;m\in \left\{\mathrm{RGB},\mathrm{Depth},\mathrm{IR}\right\},$$

where ${\bar{R}}_{m}$ denotes the aggregated response for modality *m*. This normalization ensures that the weights of the three modalities sum to one at each spatial position, allowing LCMA to dynamically adjust the relative contribution of each modality according to the input feature distribution.

**Figure 4:**
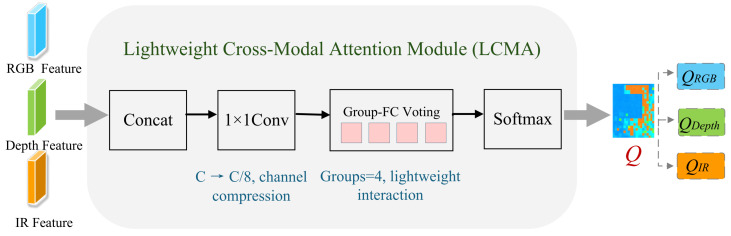
Architecture of the proposed Lightweight Cross-Modal Attention (LCMA) module.

As illustrated in Figure 4, the LCMA module first aggregates tri-modal features via channel concatenation and compression, then models cross-modal interactions using grouped 1D convolutions, finally producing the attention maps $Q=\{Q_{\mathrm{RGB}},Q_{\mathrm{Depth}},Q_{\mathrm{IR}}\}$. These attention maps act as spatially adaptive weights that enhance discriminative regions while suppressing background noise and modality-specific interference. Besides pixel-wise reweighting, the modality-aware weight maps also provide effective guidance for the subsequent cross-modal fusion in the LCMF module by highlighting spatially confident regions. Furthermore, by spatially averaging *Q*, a modality-level weight vector $\mathrm{lcma}\_\mathrm{weights}\in R^{B\times 3}$ is obtained and integrated into the LAF-Loss for modality-adaptive supervision without introducing extra learnable parameters.

Overall, LCMA achieves an explicit, dynamic, and sample-adaptive cross-modal interaction mechanism with only lightweight operations. Despite its simple structure, the module enables fine-grained modeling of modality reliability with negligible computational overhead, providing an effective basis for dynamic multimodal fusion under strict deployment constraints.

### 3.4. Lightweight Cross-Modal Fusion Guided by Spatial Weights (LCMF)

Traditional feature fusion strategies, such as direct concatenation or element-wise addition, typically rely on fixed or implicitly learned fusion weights. Such static fusion paradigms lack the ability to adapt to spatial variations and dynamic modality reliability under complex imaging conditions, which limits their effectiveness in real-world presentation attack scenarios.

Moreover, existing cross-modal fusion methods often adopt symmetric attention mechanisms or full pairwise interactions across modalities, where each modality attends to all others. Although effective, such designs introduce considerable computational overhead and redundant feature interactions, making them less suitable for lightweight deployment scenarios.

**Figure 5:**
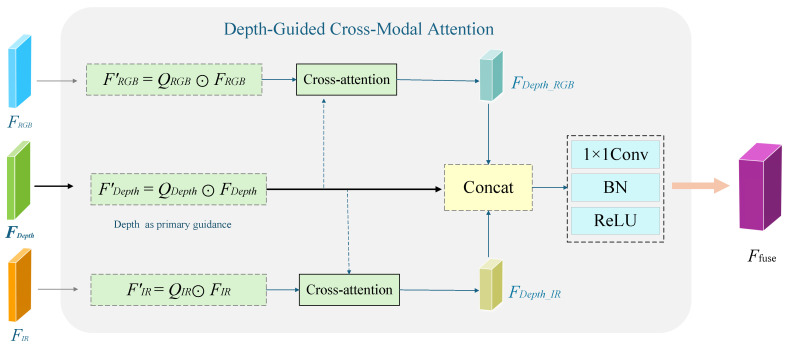
Architecture of the proposed depth-guided cross-modal fusion module.

To address these limitations, we propose a Lightweight Cross-Modal Fusion module guided by spatial weights, as illustrated in Figure 5. Guided by the spatial weight maps $Q=\{Q_{\mathrm{RGB}},Q_{\mathrm{Depth}},Q_{\mathrm{IR}}\}$ generated by the LCMA module, the proposed module performs spatially adaptive reweighting and Depth-guided semantic-level cross-modal feature reconstruction within a computationally efficient framework.

Different from conventional fusion strategies, the proposed design introduces a structurally constrained and asymmetric interaction mechanism. Specifically, Depth is treated as the anchor modality to guide cross-modal interaction, while RGB and IR features are selectively integrated. This design avoids redundant pairwise modality interactions and significantly reduces computational complexity.

At the fusion stage, features from each modality are first dynamically reweighted in a pixel-wise manner using the corresponding spatial weights:

(5) $$F_{\mathrm{Depth}}^{\prime }=Q_{\mathrm{Depth}}⊙F_{\mathrm{Depth}},\;F_{\mathrm{RGB}}^{\prime }=Q_{\mathrm{RGB}}⊙F_{\mathrm{RGB}},\;F_{\mathrm{IR}}^{\prime }=Q_{\mathrm{IR}}⊙F_{\mathrm{IR}}$$

where ⊙ denotes element-wise multiplication. This operation serves as a spatial pre-filtering mechanism, suppressing unreliable regions before cross-modal interaction, thereby reducing unnecessary computations and improving robustness.

To further capture semantic-level cross-modal correlations in a lightweight manner, the module adopts a Depth-guided attention mechanism. Specifically, the weighted Depth features are projected as the query:

(6) $$Q_{d}=W_{Q}F_{\mathrm{Depth}}^{\prime }$$

while the weighted RGB and IR features are projected as keys and values:

(7) $$K_{m}=W_{K}F_{m}^{\prime },\;V_{m}=W_{V}F_{m}^{\prime },\;m\in \left\{\mathrm{RGB},\mathrm{IR}\right\}$$

Unlike conventional multi-modal attention that models full pairwise interactions among all modalities, the proposed design only performs Depth-to-RGB and Depth-to-IR attention. As a result, the number of attention branches is reduced from three pairwise interactions to two, leading to a lower computational cost compared to conventional full pairwise attention schemes (which require three interaction branches), while preserving essential complementary information.

Using Depth as the structural anchor modality, dot-product attention is employed to dynamically model cross-modal semantic interactions and reconstruct complementary cues from RGB and IR:

(8) $$F_{\mathrm{Depth\_RGB}}=\mathrm{Softmax}\left(\frac{Q_{d}K_{\mathrm{RGB}}^{T}}{\sqrt{C}}\right)V_{\mathrm{RGB}}$$

(9) $$F_{\mathrm{Depth\_IR}}=\mathrm{Softmax}\left(\frac{Q_{d}K_{\mathrm{IR}}^{T}}{\sqrt{C}}\right)V_{\mathrm{IR}}$$

where *C* denotes the channel dimension used for attention scaling.

Finally, the reconstructed cross-modal features are concatenated with the dynamically weighted Depth features and passed through a 1×1 convolution to obtain a compact unified representation:

(10) $$F_{\mathrm{fuse}}=\psi \left(\mathrm{Concat}\left[F_{\mathrm{Depth\_RGB}},F_{\mathrm{Depth}}^{\prime },F_{\mathrm{Depth\_IR}}\right],\theta \right)$$

where ψ denotes the 1×1 convolution operation and θ represents the learnable parameters.

Through LCMA-guided spatial weighting and depth-anchored asymmetric attention reconstruction, the proposed module forms an efficient dynamic cross-modal modeling pipeline. By combining spatial pre-filtering and reduced attention interactions, the computational complexity is significantly lower than conventional full pairwise attention schemes, while still maintaining strong representation capability. This enables a favorable balance between accuracy and efficiency, making the proposed approach particularly suitable for ultra-lightweight face anti-spoofing deployment scenarios. The resulting fused feature $F_{\mathrm{fuse}}$ is subsequently fed into the lightweight classification head for final liveness prediction.

### 3.5. Decision Layer and Loss Optimization

To produce the final liveness prediction while preserving the ultra-lightweight inference property of the overall architecture, a simplified classification head is employed. It consists of a 1×1 convolution, Global Average Pooling (GAP), Dropout, and a Fully Connected (FC) layer, forming a compact yet effective decision pipeline. Given the fused feature $F_{\mathrm{fuse}}\in R^{B\times 64\times H\times W}$, a 1×1 convolution is utilized to map the channel dimension from 64 to 128:

(11) $$F_{1}={\mathrm{Conv}}_{1\times 1}\left(F_{\mathrm{fuse}}\right)\in R^{B\times 128\times H\times W}$$

Global spatial information is then aggregated via GAP:

(12) $$f_{\mathrm{gap}}=\mathrm{GAP}\left(F_{1}\right)\in R^{B\times 128}$$

After Dropout with a ratio of 0.3, the feature vector is projected into a 2-dimensional logit space through an FC layer:

(13) $$z=\mathrm{FC}\left(\mathrm{Dropout}\left(f_{\mathrm{gap}}\right)\right)\in R^{B\times 2}$$

Finally, class probabilities are obtained via Softmax:

(14) $$p=\left[p_{\mathrm{live}},p_{\mathrm{spoof}}\right]=\mathrm{Softmax}\left(z\right)$$

Beyond the compact decision head, the training objective is designed to reinforce dynamic cross-modal modeling behavior while maintaining structural efficiency. Specifically, the three following complementary loss functions are employed to jointly guide the optimization process: The Pixel-Wise Fusion Loss ($L_{\mathrm{PWL}}$) provides lightweight spatial regularization to stabilize fusion responses and suppress irrelevant activations. The Binary Cross-Entropy loss ($L_{\mathrm{BCE}}$) serves as the primary supervision for liveness classification. In addition, the proposed LCMA-guided Adaptive Fusion Loss ($L_{\mathrm{LAF}}$) dynamically adjusts modality contributions based on attention responses, encouraging the network to emphasize more reliable modalities during training.

To encourage stable and structured spatial responses in the fusion branch, a Pixel-Wise Fusion Loss ($L_{\mathrm{PWL}}$) is introduced:

(15) $$L_{\mathrm{PWL}}=\frac{1}{H\times W}\sum_{i,j}^{H\times W}{\left(Y_{i,j}-F_{i,j}\right)}^{2}$$

where *Y* denotes a target map defined according to the sample label, providing lightweight spatial supervision to suppress irrelevant activations and regularize fusion responses.

For classification supervision, the Binary Cross-Entropy loss ($L_{\mathrm{BCE}}$) is adopted:

(16) $$L_{\mathrm{BCE}}=-\frac{1}{n}\sum_{i=1}^{n}\left[y_{i}\log p_{i}+\left(1-y_{i}\right)\log\left(1-p_{i}\right)\right]$$

Furthermore, to align modality attention allocation with discriminative capability under dynamic cross-modal modeling, the LCMA-guided Adaptive Fusion Loss ($L_{\mathrm{LAF}}$) is introduced. Specifically, the attention responses generated by LCMA are used to adaptively weight modality-specific classification losses, encouraging the network to emphasize more reliable modalities during training and facilitating more effective cross-modal feature fusion. Let $p_{m}^{i}$ denote the prediction of modality m∈{RGB,Depth,IR} for sample *i*, and $y^{i}$ the corresponding ground-truth label. The modality-specific Binary Cross-Entropy loss is defined as:

(17) $$L_{m}=-\frac{1}{n}\sum_{i=1}^{n}\left[y^{i}\log p_{m}^{i}+\left(1-y^{i}\right)\log\left(1-p_{m}^{i}\right)\right]$$

LCMA produces modality attention vectors $q^{i}=\left[q_{\mathrm{RGB}}^{i},q_{\mathrm{Depth}}^{i},q_{\mathrm{IR}}^{i}\right]$, which are averaged across the batch to obtain modality weights:

(18) $$w_{m}=\frac{1}{n}\sum_{i=1}^{n}q_{m}^{i},\;m\in \left\{\mathrm{RGB},\mathrm{Depth},\mathrm{IR}\right\}$$

Since the LCMA attention responses are normalized across modalities, the resulting weights satisfy $\sum_{m}w_{m}=1$, ensuring a balanced adaptive weighting across modalities. The modality-adaptive loss is then formulated as:

(19) $$L_{\mathrm{LAF}}=\sum_{m}w_{m}L_{m}$$

The overall training objective integrates classification accuracy, spatial regularization, and modality-adaptive supervision into a unified optimization framework:

(20) $$L_{\mathrm{total}}=\lambda_{1}L_{\mathrm{BCE}}+\lambda_{2}L_{\mathrm{PWL}}+\lambda_{3}L_{\mathrm{LAF}}$$

where $\lambda_{1}$, $\lambda_{2}$, and $\lambda_{3}$ denote the balancing coefficients controlling the relative contributions of the classification objective, spatial regularization, and modality-adaptive supervision, respectively. Through this joint optimization strategy, the network learns discriminative representations while maintaining stable cross-modal fusion behavior and consistent modality attention allocation, thereby facilitating more reliable dynamic cross-modal modeling under diverse attack conditions.

### 3.6. Datasets

For all our experiments, we used three publicly available benchmark datasets: CASIA-SURF [3], CASIA-SURF CeFA [40], and CASIA-FASD [41]. All their key features are presented in Table 1 for convenient reference.

CASIA-SURF. CASIA-SURF is a large-scale multimodal dataset that is specifically designed for face anti-spoofing and presentation attack detection tasks. An important characteristic of this dataset is that it provides synchronized RGB, Depth, and Infrared data, all collected from 1000 different subjects. In total, there are approximately 21,000 video samples in the dataset. For each subject, one real (bona fide) video is paired with six attack videos which cover a wide range of different spoofing methods, making it beneficial for comprehensive evaluation. To make evaluation more standardized, the dataset is split into training, validation, and test sets. Specifically, 300 subjects are assigned to the training set, 100 to the validation set, and 600 to the test set. This split results in 148K, 48K, and 295K frames, respectively, providing sufficient diversity and robustness to train and evaluate the model effectively.

CASIA-SURF CeFA. CASIA-SURF CeFA is an extension of the original CASIA-SURF dataset, and the main addition is the inclusion of cross-ethnicity considerations. This makes it a comprehensive benchmark, not only for multimodal face anti-spoofing but also for fairness analysis. Similar to the original dataset, it includes RGB, Depth, and Infrared modalities, and it covers subjects from three ethnic groups: African, East Asian, and Central Asian. There are also two attack subsets included. The 2D subset contains samples from 1500 subjects—4500 bona fide samples and 13,500 attack samples. As for the 3D subset, it contains 5538 attack videos collected from 107 subjects; these videos are captured under different illumination conditions, which increases the diversity of the dataset. To thoroughly evaluate model generalization, the dataset defines four evaluation protocols, including Protocol 1 for cross-ethnicity evaluation, Protocol 2 for cross-PAI evaluation, Protocol 3 for cross-modality evaluation, and Protocol 4, which combines cross-ethnicity and cross-PAI settings. Each of these protocols is further divided into multiple sub-protocols for more detailed assessment. In our study, we focus on Protocol 4 and its sub-protocols because they represent the most challenging cross-domain scenarios. All protocols use subject-independent splits: 200 subjects for training, 100 for validation, and 200 for testing.

CASIA-FASD. CASIA-FASD is one of the most widely used datasets for face anti-spoofing, although it only contains RGB data (without Depth or Infrared modalities). It includes video recordings from 50 subjects, and each subject provides 12 video clips covering both real and spoofed samples. The videos are captured at three different quality levels: low, medium, and high. They also include three common attack types: warped photo attacks, cut photo attacks, and video replay attacks. Because it contains a wide variety of attack patterns and capture conditions, CASIA-FASD remains a classic benchmark for evaluating how well face anti-spoofing methods generalize to different scenarios.

### 3.7. Evaluation Metrics

We adopt a unified evaluation protocol to comprehensively assess both the effectiveness and efficiency of the proposed multimodal face anti-spoofing framework. Detection performance is primarily evaluated using the Attack Presentation Classification Error Rate (APCER) and the Normal Presentation Classification Error Rate (NPCER), which measure the misclassification rates of spoofed and genuine samples, respectively. Their average defines the Average Classification Error Rate (ACER), which is widely used as an overall performance indicator. However, it is important to note that ACER assigns equal weight to APCER and NPCER and does not explicitly account for the inherent class imbalance in the CASIA-SURF dataset (approximately 1:6 ratio between genuine and spoof samples). As a result, ACER may obscure asymmetric error distributions and potentially provide an optimistic estimation of model performance. To provide a more transparent and reliable evaluation, we additionally report APCER and NPCER separately, allowing a detailed analysis of the model’s behavior with respect to different types of errors. Beyond these metrics, we further evaluate system performance under practical deployment constraints by reporting the True Positive Rate at fixed False Positive Rate (TPR@FPR). Specifically, we consider FPR values of $10^{-2}$, $10^{-3}$, and $10^{-4}$, corresponding to low-, medium-, and high-security application scenarios, respectively. In particular, the FPR = $10^{-4}$ setting reflects strict access control environments, where only a very limited number of false acceptances can be tolerated, thus providing a meaningful operational reference. In addition, we include the Equal Error Rate (EER) as a threshold-independent metric to evaluate the trade-off between false acceptance and false rejection. EER is defined as the error rate at the operating point where the False Positive Rate (FPR) equals the False Negative Rate (FNR). Since an exact equality is rarely achieved in practice, EER is computed at the threshold that minimizes the difference between these two error rates.We also report the Half Total Error Rate (HTER), which is equivalent to ACER but widely adopted in general biometric recognition literature for consistent comparison. On the efficiency side, we report parameter count (Params) to measure model size and floating-point operations (FLOPs) to quantify computational cost. Together, these metrics provide a comprehensive evaluation of the trade-off between detection accuracy and computational efficiency.

(21) $$\mathrm{TPR}=\frac{\mathrm{TP}}{\mathrm{TP}+\mathrm{FN}}.$$

(22) $$\mathrm{FPR}=\frac{\mathrm{FP}}{\mathrm{FP}+\mathrm{TN}}.$$

(23) $$\mathrm{FNR}=\frac{\mathrm{FN}}{\mathrm{TP}+\mathrm{FN}}.$$

(24) $$\mathrm{APCER}=\frac{\mathrm{FP}}{\mathrm{FP}+\mathrm{TN}}.$$

(25) $$\mathrm{NPCER}=\frac{\mathrm{FN}}{\mathrm{TP}+\mathrm{FN}}.$$

(26) $$\mathrm{ACER}=\frac{\mathrm{APCER}+\mathrm{NPCER}}{2}.$$

(27) $$\tau^{*}=\arg\min_{\tau }\left|\mathrm{FPR}(\tau )-\mathrm{FNR}(\tau )\right|.$$

(28) $$\mathrm{EER}=\mathrm{FPR}\left(\tau^{*}\right)=\mathrm{FNR}\left(\tau^{*}\right).$$

(29) $$\mathrm{HTER}=\frac{\mathrm{APCER}+\mathrm{NPCER}}{2}.$$

### 3.8. Implementation Details

All our code is implemented in PyTorch 1.9.0 with Python 3.8. We run the training and evaluation on a workstation with an NVIDIA RTX 4090D GPU (24 GB memory) and an Intel Xeon Platinum 8474C CPU. During training, we apply standard data augmentation—random horizontal flipping, rotation, and cropping—to help the model handle variations in appearance. Inputs are resized to 64×64, which we found gives a good balance between accuracy and speed. The network is optimized using stochastic gradient descent (SGD) with a momentum parameter of 0.9 and a weight decay coefficient of $5\times 10^{-4}$. The learning rate starts at 0.01 and follows a cosine annealing schedule. We fix the batch size at 32 across all runs. Depending on the dataset, training runs for 1 to 10 cycles, each lasting 100 epochs. In multimodal experiments, we use a joint loss that handles classification, spatial regularization, and modality adaptation simultaneously, with weights set to $\lambda_{1}=0.4$, $\lambda_{2}=0.6$, and $\lambda_{3}=0.3$. For simpler single-modal setups, we scale this back to $\lambda_{1}=0.8$, $\lambda_{2}=0$, and $\lambda_{3}=0$, which we found keeps training stable and helps it converge faster.

## 4. Results Analysis

### 4.1. Validity Analysis of the Proposed Model

To examine the effectiveness of the proposed dynamic cross-modal modeling framework under an ultra-lightweight architecture, a series of experiments are carried out on the CASIA-SURF dataset from three complementary perspectives, namely input resolution, modality configuration, and feature fusion strategy. Performance is evaluated using the metrics introduced in Section 4.2, including ACER, APCER, NPCER, and TPR@FPR. In addition, computational overhead is measured by floating-point operations (FLOPs) and the overall parameter count (Params), allowing a joint evaluation of detection accuracy and deployment efficiency. First, the influence of input resolution is investigated by testing five input sizes (32×32, 48×48, 64×64, 80×80, and 96×96) under identical network architecture and training settings. As reported in Table 2, the input resolution of 64×64 provides the best trade-off between accuracy and efficiency, preserving sufficient facial structural information while maintaining low computational overhead. Therefore, 64×64 is adopted in all subsequent experiments. Second, the impact of different modality combinations is analyzed, including single-modality (RGB, Depth, IR), dual-modality (RGB–Depth, RGB–IR, Depth–IR), and tri-modal (RGB–Depth–IR) inputs. As shown in Table 3, multimodal configurations consistently outperform single- and dual-modality settings across all evaluation metrics, indicating that dynamic cross-modal modeling effectively exploits complementary information among RGB, Depth, and IR modalities. Finally, three fusion strategies—namely, input-level fusion, score-level fusion, and feature-level fusion—are compared within the same backbone and dynamic cross-modal modeling framework. This setup ensures a fair comparison by isolating the fusion mechanism as the sole experimental variable, with corresponding results presented in Table 4. It is worth noting that all fusion strategies are evaluated under the same network configuration, and the observed performance differences are solely attributed to the effectiveness of the fusion mechanism. Feature-level fusion achieves the best overall performance, demonstrating that adaptive fusion in the deep feature space is more sui for dynamic cross-modal interaction modeling than static fusion paradigms. Although the absolute improvement in ACER appears numerically small, it corresponds to a substantial relative reduction of approximately 74% and 65% compared to input-level and score-level fusion, respectively. Moreover, consistent improvements across all TPR@FPR metrics further confirm the robustness and consistency of the feature-level fusion strategy.

Overall, these results validate the effectiveness and robustness of the proposed dynamic cross-modal modeling framework while confirming its favorable balance between performance and computational efficiency, thereby providing a solid foundation for the subsequent ablation studies and cross-dataset evaluations.

### 4.2. Ablation and Benchmark Experiments on CASIA-SURF

#### 4.2.1. Module Ablation Study

To evaluate the effectiveness of the proposed framework under ultra-lightweight constraints, we conduct a progressive module-by-module ablation study on CASIA-SURF. Starting from ShuffleNetV2_0.5 as the baseline, GGS-BlockA, SE, LCMA, and LCMF are sequentially integrated to validate their individual contributions. The results are reported in Table 5.

As shown, the ACER is consistently reduced from 0.9528% to 0.0640% as components are progressively added, demonstrating a clear performance improvement path. The introduction of GGS-BlockA significantly enhances feature representation while substantially reducing parameters and computational cost, establishing an efficient lightweight backbone. The SE module further improves channel-wise feature discrimination with negligible overhead. Subsequently, incorporating cross-modal modeling modules (LCMA and LCMF) leads to further performance gains, indicating the benefit of exploiting complementary information across modalities. The final model achieves the best performance with only 0.1441 M parameters and 0.00650 G FLOPs, highlighting a favorable trade-off between accuracy and efficiency.

Overall, the progressive ablation clearly demonstrates that the proposed design achieves steady and significant performance gains while maintaining strict ultra-lightweight constraints, further validating its practicality for real-world edge deployment.

#### 4.2.2. Leave-One-Out Ablation Study

To complement the progressive ablation, we further conduct a leave-one-out study to assess the individual contribution of each component under a controlled setting. Specifically, each module is removed from the full model while keeping the remaining structure intact. Since LCMF depends on the modality-aware weights generated by LCMA, removing LCMA requires replacing LCMF with a static fusion strategy to ensure a valid and fair comparison. The results are reported in Table 6. Removing any component leads to consistent performance degradation across ACER, APCER, NPCER, and TPR@FPR metrics, confirming that all modules contribute positively to the overall framework. Notably, removing LCMA results in the most significant degradation, with clear increases in both APCER and NPCER and corresponding drops in TPR@FPR. This demonstrates that dynamic modality reweighting is essential for effectively handling both attack and bona fide samples, thereby validating the core design motivation. In contrast, removing LCMF causes a moderate performance drop, indicating its complementary role in enhancing cross-modal feature fusion. The relatively smaller impact of removing SE suggests that channel-wise recalibration provides auxiliary benefits, while the degradation observed without GGS-BlockA highlights the importance of the optimized lightweight backbone.

Overall, the leave-one-out analysis provides complementary and more rigorous evidence beyond progressive ablation, confirming that each component is independently necessary and effectively integrated into the proposed LAM-FAS framework.

#### 4.2.3. Benchmark Experiments and Joint Loss Optimization

Following the module-level ablation study, a comprehensive benchmark evaluation is conducted to assess the effectiveness of the proposed dynamic cross-modal modeling framework under realistic ultra-lightweight deployment constraints. For fair comparison, all re-implemented methods are trained under identical experimental settings with the same multimodal input configuration. Our approach is evaluated against representative lightweight backbone networks, including MobileNetV2, ShuffleNetV2, and GhostNet, as well as competitive multimodal face anti-spoofing methods such as FaceBagNet and models from the FeatherNet family. The detailed quantitative results are presented in Table 7.

As shown in Table 7, the proposed method achieves consistently strong detection performance while maintaining an extremely compact model size and low computational cost. For completeness, we also include the halfway fusion baseline from the CASIA-SURF benchmark, which adopts a multi-stream ResNet-18 backbone with a simple intermediate fusion strategy. This baseline helps to illustrate the impact of fusion design on multi-modal face anti-spoofing performance. Although some existing approaches, such as FaceBagNet and FeatherNet, report competitive results, they often rely on multi-branch architectures or high-dimensional feature representations, which increase structural complexity and computational overhead in practical deployment scenarios. In contrast, the proposed framework is built upon an ultra-lightweight backbone and introduces only a small number of low-dimensional cross-modal interaction modules to enable explicit and adaptive dynamic modeling. As a result, the model remains within a strict lightweight budget (0.14 M parameters and 0.0065 G FLOPs) while significantly enhancing multimodal feature discrimination capability. This favorable performance–efficiency balance makes the framework particularly suitable for edge-oriented face anti-spoofing applications. Quantitatively, the proposed method achieves an ACER of 0.064%, outperforming several representative baselines, including MA-Net (2.00%), MMFCNN (1.13%), FeatherNet (0.1292%), and FaceBagNet (0.0985%). Moreover, under increasingly strict false positive rate constraints (TPR@FPR of $10^{-2}$, $10^{-3}$, and $10^{-4}$), the detection rates remain consistently high at 99.9750%, 99.9322%, and 99.6608%, respectively, demonstrating s discrimination capability in high-security scenarios. To ensure a fair and consistent comparison, several mainstream lightweight architectures—MobileNetV2, GhostNet, FeatherNetA/B, and ShuffleNetV2—are re-implemented within the same experimental framework using identical multimodal inputs, network width configurations, and training strategies. The results marked as “in our” in 6 correspond to these reproduced baselines. Although some reproduced models exhibit slightly fewer parameters or FLOPs due to their simpler structures, they lack explicit cross-modal interaction modeling. Consequently, their ability to handle complex and diverse presentation attack patterns is comparatively limited.

By incorporating adaptive cross-modal attention and fusion mechanisms while strictly controlling computational complexity, the proposed method achieves superior feature alignment and modality complementarity without sacrificing efficiency. Overall, the benchmark results indicate that the proposed dynamic cross-modal modeling framework maintains a favorable trade-off between detection accuracy, robustness, and lightweight deployment efficiency.

### 4.3. Protocol-Based Evaluation on the CASIA-SURF CeFA Dataset

To further evaluate the generalization capability and deployment robustness of the proposed dynamic cross-modal modeling framework, protocol-driven experiments are conducted on the CASIA-SURF CeFA dataset, with detailed results reported in Table 8. Compared with conventional benchmarks, CASIA-SURF CeFA exhibits substantially higher variability in sensing devices, illumination conditions, capture environments, subject demographics, and presentation attack types. As such, it is widely recognized as a rigorous benchmark for assessing cross-domain robustness in face anti-spoofing.

All experiments strictly follow the official evaluation protocols of CASIA-SURF CeFA to ensure fair and consistent comparison. In particular, Protocol 4 and its three sub-protocols—namely, Protocol 4-1, Protocol 4-2, and Protocol 4-3—simultaneously consider cross-device, cross-scene, and cross-attack settings, making them among the most challenging evaluation scenarios in the dataset. These protocols closely resemble real-world deployment environments, where distribution shifts and heterogeneous attack patterns are inevi. Therefore, our analysis primarily focuses on Protocol 4 and its related sub-protocols. As summarized in Table 8, the proposed method maintains consistently low APCER, NPCER, and ACER across all evaluated sub-protocols, demonstrating s cross-domain detection capability. Under Protocol 4-2, although the proposed method achieves the lowest ACER (0.78%), a certain degree of asymmetry between APCER (1.32%) and NPCER (0.24%) can be observed. This phenomenon is not unique to our method but is also evident in other approaches, such as PipeNet, which exhibits a larger discrepancy (APCER 4.11% vs. NPCER 1.50%). This suggests that error imbalance is a common challenge under complex cross-domain conditions, where variations in attack types and sensing environments may unevenly affect different error categories. Compared with these methods, the proposed approach maintains a relatively lower overall error level while reducing the imbalance between APCER and NPCER, indicating a more favorable trade-off between spoof rejection and genuine acceptance. Furthermore, under Protocol 4-3, the proposed method achieves a more balanced error distribution (APCER 1.84% vs. NPCER 1.28%), further demonstrating its ability to adapt to diverse and heterogeneous scenarios. These observations suggest that the proposed dynamic cross-modal modeling mechanism not only improves overall detection performance, but also enhances robustness by mitigating extreme bias toward specific error types.

### 4.4. Single-Modal Evaluation on the CASIA-FASD Dataset

To further evaluate the intrinsic representation capability of the proposed ultra-lightweight architecture, we perform additional experiments on the CASIA-FASD dataset, and the detailed results are presented in Table 9. Unlike the previously evaluated multimodal benchmarks, CASIA-FASD provides only RGB data, which more closely reflects practical deployment scenarios where auxiliary sensors such as Depth or Infrared are unavailable due to hardware or cost constraints. This setting enables evaluation of the backbone’s standalone discriminative capacity without relying on dynamic cross-modal supervision. Under the single-modality configuration, only the RGB branch and its corresponding feature extraction and classification components are retained, while all cross-modal attention and fusion modules are removed. Therefore, the reported results primarily reflect the effectiveness of the proposed lightweight backbone in capturing appearance-driven liveness cues using RGB inputs alone. As shown in Table 9, the proposed method achieves EER and HTER values of 1.08% and 1.27%, respectively, which are comparable to or better than several existing approaches. These results indicate that the ultra-lightweight architecture maintains strong discrimination capability even without multimodal interaction modeling. Beyond numerical performance, this evaluation further demonstrates the structural robustness and adaptability of the proposed framework across different modality configurations. The model remains stable and reliable in single-modality settings, confirming that the dynamic cross-modal modules enhance performance when available, while the core lightweight backbone itself remains effective and deployment-ready under resource-constrained conditions.

### 4.5. Robustness Under Missing Modalities

To further evaluate the robustness of LAM-FAS under practical deployment scenarios where certain modalities may be unavailable, we conduct experiments under missing modality conditions on the CASIA-SURF dataset. Following a commonly adopted missing modality evaluation protocol [19], the model is trained with full RGB + Depth + IR modalities and tested with incomplete modality combinations.

Four testing protocols are considered:

- P1: RGB-only (both Depth and IR missing).
- P2: RGB + Depth (IR missing).
- P3: RGB + IR (Depth missing).
- P4: RGB + Depth + IR (full modalities, baseline).

The evaluation metric is ACER (%), where lower values indicate better performance. As shown in Table 10, the performance of LAM-FAS decreases progressively as modalities are removed during inference. The full-modal setting (P4) achieves the best performance with an ACER of 0.0640%. When the IR modality is missing (P3), the ACER increases to 0.4126%, representing a moderate performance decrease. In the absence of the Depth modality (P2), the ACER rises to 0.1852%, indicating a more substantial degradation. In the extreme case where only RGB is available (P1), the ACER increases sharply to 2.8640%, consistent with the known limitations of single-modality FAS systems. These results demonstrate that LAM-FAS effectively leverages complementary information across modalities while maintaining reasonable robustness under missing modality conditions. Moreover, this behavior aligns with prior studies on cross-modal feature transitions, which indicate that appropriate fusion and attention mechanisms can partially mitigate the impact of missing modalities [19]. Overall, the findings highlight the inherent dependence of LAM-FAS on multi-modal inputs for optimal performance.

### 4.6. Cross-Dataset Generalization

To further verify the generalization ability of the proposed LAM-FAS in real cross-scenario applications, we conduct cross-dataset evaluation between CASIA-SURF and CASIA-SURF CeFA. Following the standard cross-domain protocol, the model is trained on one dataset and directly tested on the other without any fine-tuning or domain adaptation. These two datasets present substantial domain gaps in terms of attack types, illumination conditions, subject ethnicity, and acquisition protocols, providing a sufficiently challenging cross-domain scenario to effectively evaluate the generalization capability of LAM-FAS. The results are listed in Table 11. Due to the obvious domain gap in illumination, attack types, sample distribution, and acquisition conditions, the performance decreases naturally compared with intra-dataset tests. However, LAM-FAS still maintains stable and competitive cross-dataset performance, with ACER values of 5.32% and 3.87% in the two cross-test settings. Furthermore, we observe that the model trained on CASIA-SURF CeFA performs better (3.87%) than the one trained on CASIA-SURF (5.32%). This is because CASIA-SURF CeFA contains more diverse attacks, cross-ethnicity subjects, and complex illumination conditions, leading to a more generalized and robust feature space. In contrast, the simpler and more homogeneous CASIA-SURF dataset provides less diversity for cross-domain generalization. This observation indicates that the proposed dynamic cross-modal modeling and lightweight feature learning scheme can effectively capture modality-invariant and domain-robust representations, rather than merely memorizing dataset-specific biases. The satisfactory cross-dataset performance further validates the practical deployment potential of LAM-FAS under realistic and unseen environments.

### 4.7. Deployment Efficiency on Edge Devices

To verify the real-world deployability of the proposed LAM-FAS on resource-constrained hardware, we evaluate its inference efficiency on the NVIDIA Jetson Nano (4 GB), a widely used single-board computer for edge computing. In contrast to the theoretical complexity metrics (i.e., Params and FLOPs) reported in previous sections, here we measure three practical deployment indicators: inference latency, frame rate (FPS), and runtime memory usage. This demonstrates that, although modern SBCs can run larger models, achieving low-latency real-time performance in latency-sensitive applications still necessitates a highly efficient, ultra-lightweight architecture like LAM-FAS. As summarized in Table 12, LAM-FAS achieves the best performance among all compared lightweight face anti-spoofing models. Specifically, our method obtains the lowest latency of 11.2 ms, the highest throughput of 89.3 FPS, and an efficient memory footprint of 36.7 MB. It is widely acknowledged that typical lightweight models deployed on the Jetson Nano usually achieve 15–30 FPS for real-time face recognition applications, while larger models often run below 10 FPS [5,21,35]. In contrast, LAM-FAS far exceeds the real-time requirement, making it highly suitable for latency-sensitive embedded scenarios such as access control, mobile payment, and smart surveillance. These results demonstrate that the proposed ultra-lightweight architecture not only reduces theoretical computational overhead but also delivers excellent practical inference efficiency on real edge devices, which strongly validates our original design motivation for resource-constrained deployment.

### 4.8. Visualization and Interpretability Analysis

**Figure 6:**
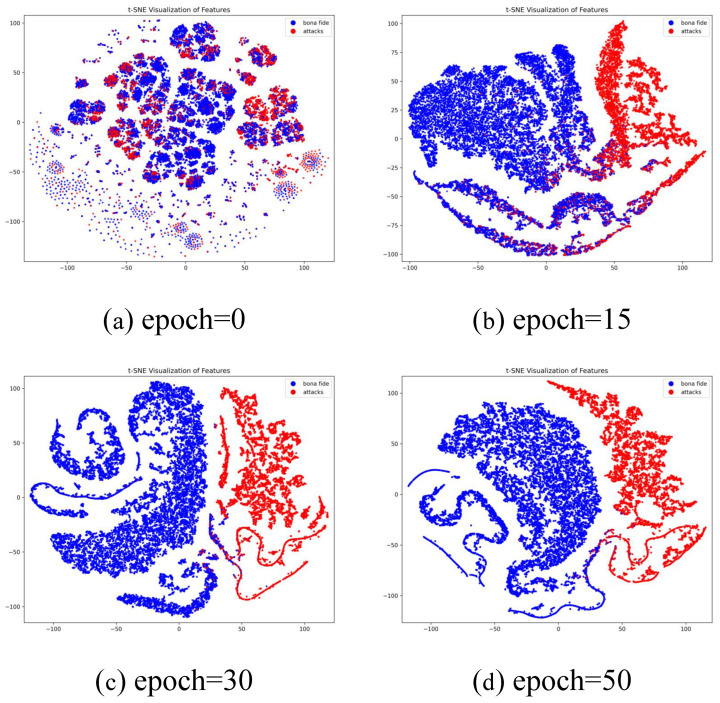
t-SNE Visualization of Feature Representations at Different Training Epochs on CASIA-SURF.

To provide a deeper understanding of how discriminative representations are progressively constructed under the proposed dynamic cross-modal modeling framework, t-SNE [48] is employed to visualize the feature distributions of bona fide and spoof samples on the CASIA-SURF dataset. Feature embeddings extracted at representative training stages (Epoch=0,15,30,50) are selected to trace the evolution of the learned feature space, as illustrated in Figure 6. This staged visualization allows us to examine not only the final separability but also the dynamic formation process of class-discriminative structures.

At Epoch=0, the network parameters are randomly initialized, and the resulting feature embeddings are widely scattered with substantial overlap between bona fide and spoof samples. No meaningful clustering structure can be observed, indicating that the model has not yet captured appearance-based or cross-modal discriminative cues. This initial state serves as a baseline, reflecting the intrinsic difficulty of separating real and spoof faces without learned representation guidance. As training progresses to Epoch=15, the feature space begins to exhibit early organizational patterns. Although inter-class overlap remains, loosely formed clusters start to emerge, suggesting that the network has started to identify preliminary liveness-related cues. Notably, the separation at this stage is not abrupt but gradual, implying that the dynamic modeling mechanism incrementally adjusts feature interactions rather than enforcing overly aggressive separation. This behavior is particularly important for preventing unstable convergence under lightweight architectural constraints. By Epoch=30, the embeddings become significantly more compact within each class, and the margin between bona fide and spoof clusters becomes increasingly distinguishable. The reduction in ambiguous overlapping regions indicates that cross-modal interactions and adaptive feature refinement are effectively enhancing discriminative consistency. The progressive tightening of intra-class distributions suggests that the model is not merely memorizing superficial cues, but rather learning structured and semantically coherent representations. At Epoch=50, the feature space appears largely stabilized, characterized by strong intra-class cohesion and clear inter-class separation. Only a limited number of boundary samples remain near the decision margin, which is expected given the intrinsic complexity of certain attack types. The smooth and monotonic evolution from scattered distributions to well-structured clusters demonstrates that the proposed ultra-lightweight architecture can dynamically refine representations throughout training without relying on excessive depth or parameter redundancy.

Overall, this visualization analysis reveals two key properties of the proposed framework. First, the feature separation emerges progressively and stably, reflecting effective dynamic modeling rather than abrupt optimization behavior. Second, despite its compact design, the architecture achieves well-organized embedding structures comparable to heavier models. Such interpretable and stable feature evolution further substantiates the robustness, convergence reliability, and deployment suitability of the proposed method for real-world, resource-constrained face anti-spoofing scenarios.

### 4.9. Modality Contribution Analysis

To further validate the core assumption of the proposed LCMA module, we analyze the modality-level contributions under different attack types. Specifically, the modality weights are obtained by spatially averaging the attention maps $Q=\{Q_{\mathrm{RGB}},Q_{\mathrm{Depth}},Q_{\mathrm{IR}}\}$ generated by LCMA, where the weights are normalized via Softmax and thus sum to one.

**Figure 7:**
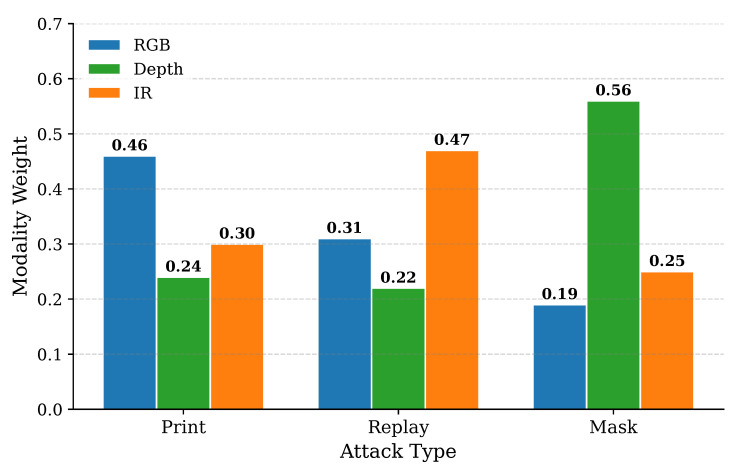
Visualization of LCMA-derived modality contributions for different attack types on CASIA-SURF CeFA. Each bar represents the normalized weight of RGB, Depth, and IR modalities.

The visualization results on the CASIA-SURF CeFA dataset are shown in Figure 7. It can be observed that the dominant modality varies across different attack scenarios. In particular, RGB contributes more under print attacks due to its sensitivity to texture cues, while IR becomes more informative for replay attacks by capturing display-related artifacts. For mask attacks, depth plays the most critical role as it provides reliable 3D structural information. These observations indicate that modality importance is not fixed but highly dependent on the attack type and input characteristics. Therefore, a static or depth-dominant fusion strategy is insufficient to handle diverse spoofing patterns. In contrast, the proposed LCMA enables dynamic and input-adaptive modality reweighting, which provides a more flexible and effective mechanism for cross-modal feature interaction.

Overall, this analysis provides empirical evidence supporting the necessity of dynamic modality modeling and justifies the design of the LCMA module. Notably, Depth does not consistently dominate across all attack types, providing direct evidence against a fixed depth-biased strategy.

## 5. Limitations and Future Work

One limitation of the proposed LAM-FAS lies in its dependency on complete multi-modal inputs during inference. As shown in Table 10, the detection performance degrades when Depth or IR modalities are absent, with a noticeable increase in ACER under partial or single-modality settings. This behavior indicates that the current dynamic cross-modal modeling strategy, while effective under full-modality conditions, is still sensitive to modality incompleteness. From a modeling perspective, the LCMA and LCMF modules rely on the availability of complementary cross-modal cues to perform reliable feature reweighting and fusion. When certain modalities are missing, the learned cross-modal interactions become less informative, leading to suboptimal modality alignment and degraded representation quality. This limitation is consistent with prior studies on cross-modal feature transitions [19], which highlight the challenges of maintaining robust performance under incomplete modality scenarios. In real-world applications, such conditions may arise due to sensor failure, environmental interference, or hardware constraints. Therefore, improving robustness to missing modalities remains an important direction for future work. Potential solutions include incorporating cross-modal knowledge distillation, designing modality-agnostic feature representations, and developing adaptive inference strategies that can dynamically compensate for missing inputs.

## 6. Conclusions

This work re-examines several critical challenges that hinder the practical deployment of face anti-spoofing systems, including modality inconsistency, inflexible fusion strategies, and strict computational constraints. To address these issues, we propose a dynamic cross-modal modeling framework built upon an ultra-lightweight architecture. Rather than increasing model depth or parameter redundancy, the proposed design emphasizes adaptive cross-modal interaction within a compact backbone, enabling effective exploitation of complementary multimodal cues while maintaining low computational overhead.

Extensive experiments on public benchmarks validate the effectiveness and robustness of the proposed framework. On multimodal datasets such as CASIA-SURF and CASIA-SURF CeFA, the model achieves stable and competitive performance under diverse capture conditions and challenging cross-domain scenarios, demonstrating strong adaptive multimodal reasoning capability. Meanwhile, single-modal evaluation on CASIA-FASD confirms that the lightweight backbone preserves reliable discriminative power even in the absence of auxiliary modalities, highlighting structural stability and deployment flexibility. Furthermore, visualization analysis reveals a smooth and progressive evolution of feature representations during training, indicating stable convergence and effective dynamic refinement within a compact model structure. Overall, the proposed method achieves a balanced trade-off among detection accuracy, adaptive cross-modal modeling, and computational efficiency, making it well suited for real-time face anti-spoofing in edge and resource-constrained environments. Future work will further investigate robustness under more severe cross-domain shifts and unseen presentation attack patterns to enhance long-term generalization and practical reliability.

## Figures and Tables

**Table 1:** Experimental datasets.

Dataset	Year	Number of Subjects	Number of Samples	Attack Types	Modal
CASIA-SURF	2018	1000	21,000 (V)	Print, Cut	RGB/Depth/IR
CASIA-SURF CeFA	2019	1607	23,538 (V)	Print, Replay, Mask	RGB/Depth/IR
CASIA-FASD	2012	50	600 (V)	Print, Replay	RGB

**Table 2:** Impact of Input Resolution on Model Performance on CASIA-SURF.

Patch Size	ACER (%)	TPR (%)@FPR=		
		10^−2^	10^−3^	10^−4^
32 × 32	1.5191	97.0828	91.3161	83.1072
48 × 48	0.4626	99.9322	98.6431	92.8765
64 × 64	**0.0640**	**99.9750**	**99.9322**	**99.8643**
80 × 80	0.2706	99.8643	99.6608	99.4573
96 × 96	0.8452	98.1004	91.0448	88.8060

**Table 3:** Performance Comparison under Different Modality Combinations on CASIA-SURF.

Modals	ACER (%)	TPR (%)@FPR=		
		10^−2^	10^−3^	10^−4^
RGB	6.7800	71.3026	32.8358	17.2320
Depth	0.5256	99.9322	97.4220	86.3636
IR	2.8713	88.8738	68.0461	58.4125
RGB + Depth	0.4217	99.7965	98.6431	95.1153
RGB + IR	0.7763	99.5929	98.7788	88.1954
Depth + IR	0.2972	99.9322	99.1180	96.6757
RGB + Depth + IR(Ours)	**0.0640**	**99.9750**	**99.9322**	**99.8643**

**Table 4:** Comparison of Different Feature Fusion Strategies on CASIA-SURF.

Fusion Mode	ACER (%)	TPR (%)@FPR=		
		10^−2^	10^−3^	10^−4^
Input-level fusion	0.2482	99.8643	99.5251	98.9145
Score-level fusion	0.1808	99.8865	99.7286	98.9824
Feature-level fusion (Ours)	**0.0640**	**99.9750**	**99.9322**	**99.8643**

**Table 5:** Ablation Study of Key Components in the Proposed LAM-FAS on CASIA-SURF.

Model	ACER (%)	TPR (%)@FPR=			Params (M)	FLOPs (G)
		10^−2^	10^−3^	10^−4^		
ShuffleNetV2_0.5 × (Baseline)	0.9528	99.3894	96.8114	95.3867	1.0325	0.09580
Improved Backbone (GGS-BlockA)	0.2336	99.7965	97.2863	96.3894	0.2463	0.01050
Improved Backbone + SE	0.1846	99.9346	99.8643	99.5929	0.2471	0.01012
Improved Backbone + SE + LCMA	0.0979	99.9568	99.8643	99.7965	0.1451	0.01015
Improved Backbone + SE + LCMF	0.0941	99.9632	99.8652	99.7622	0.1441	0.01032
LAM-FAS (Ours)	**0.0640**	**99.9750**	**99.9322**	**99.8643**	**0.1441**	**0.00650**

**Table 6:** Leave-one-out Ablation Study of LAM-FAS on CASIA-SURF.

Model	ACER (%)	APCER (%)	NPCER (%)	TPR (%)@FPR=		
				10^−2^	10^−3^	10^−4^
LAM-FAS (Full Model)	**0.0640**	**0.0603**	**0.0677**	**99.9750**	**99.9322**	**99.8643**
w/o GGS-BlockA	0.1284	0.1351	0.1217	99.8423	99.5214	98.9736
w/o SE	0.0827	0.0795	0.0859	99.9216	99.7032	99.2158
w/o LCMA	0.1612	0.1685	0.1539	99.7684	99.2147	98.4362
w/o LCMF	0.1036	0.1092	0.0980	99.8651	99.4638	98.9574

**Table 7:** Performance Comparison with State-of-the-Art Methods on CASIA-SURF.

Methods	ACER (%)	TPR (%)@FPR=			Params (M)	FLOPs (G)
		10^−2^	10^−3^	10^−4^		
Baseline [3]	2.4000	96.7000	81.8000	56.8000	—	—
MMFCNN [13]	1.1300	98.7000	91.1000	76.1000	—	—
MA-Net [24]	2.0000	96.0000	82.6000	58.1000	—	—
Wang et al. [23]	0.7000	99.5000	81.5000	55.8000	—	—
FaceBagNet [12]	0.0985	100.0000	99.9472	99.8052	—	—
FeatherNet [5]	0.1292	99.9541	99.8396	98.1441	—	—
Halfway fusion (ResNet18) [3]	4.7000	89.1000	33.6000	17.8000	—	—
FeatherNetA [5] (in our)	0.2297	99.8643	99.5251	99.3894	0.14	0.0065
FeatherNetB [5] (in our)	0.2109	99.9322	99.6608	99.2537	2.95	0.0288
MobileNetV2 [26] (in our)	1.4108	98.7788	95.7259	90.7734	0.96	0.0218
GhostNet [29] (in our)	0.3195	99.8500	99.5929	99.0502	1.45	0.0351
ShuffleNetV2 [27] (in our)	0.1731	99.9322	99.7286	95.9973	0.14	0.0147
LAM-FAS (Ours)	**0.0640**	99.9750	99.9322	99.6608	**0.14**	**0.0065**

**Table 8:** Protocol-Based Performance Comparison with Existing Methods on the CASIA-SURF CeFA Dataset.

Protocol	Method	APCER (%)	NPCER (%)	ACER (%)
Protocol 4-1	PSMM-Net [40]	5.0	3.3	4.2
	CDCN [42]	0.33	0.5	0.42
	PipeNet [37]	1.44	0.00	0.72
	LAM-FAS (Ours)	0.64	0.56	0.60
Protocol 4-2	PSMM-Net [40]	7.7	9.0	8.4
	CDCN [42]	1.39	0.75	1.07
	PipeNet [37]	4.11	1.50	2.80
	LAM-FAS (Ours)	**1.32**	**0.24**	**0.78**
Protocol 4-3	PSMM-Net [40]	10.8	4.3	7.6
	CDCN [42]	1.44	1.75	1.6
	PipeNet [37]	2.55	2.24	2.40
	LAM-FAS (Ours)	1.84	**1.28**	**1.56**

**Table 9:** Single-Modal Performance Comparison with Existing Methods on the CASIA-FASD Dataset.

Methods	EER (%)	HTER (%)
Arora et al. [43]	-	8.79
LSTM-CNN [44]	5.17	5.93
Patch and depth [8]	2.67	2.27
DPCNN [45]	4.50	-
Sun et al. [46]	1.85	1.48
CNN and SWLD [47]	2.14	2.62
LAM-FAS (Ours)	**1.08**	**1.27**

**Table 10:** Performance of LAM-FAS under missing modality conditions on CASIA-SURF. Evaluation metric: ACER (%) ↓. The model is trained with full three modalities.

Method	P1: RGB	P2: RGB + D	P3: RGB + IR	P4: RGB + D + IR
LAM-FAS (Ours)	2.8640	0.1852	0.4126	**0.0640**

**Table 11:** Cross-dataset generalization performance of LAM-FAS. The model is trained on the source dataset and directly tested on the target dataset without fine-tuning. Evaluation metric is ACER (%) ↓.

Training Dataset	Testing Dataset	ACER (%)
CASIA-SURF	CASIA-SURF CeFA	5.32
CASIA-SURF CeFA	CASIA-SURF	3.87

**Table 12:** Deployment efficiency comparison on NVIDIA Jetson Nano (4GB). Latency: average inference time per frame; FPS: frames per second; All models are reimplemented and tested under the same environment (input size 64×64, batch size 1, PyTorch).

Model	Latency (ms)	FPS	Memory (MB)
MobileNetV2-based FAS	41.8	23.9	146.2
ShuffleNetV2-based FAS	18.6	53.7	48.5
FeatherNet-B	56.3	17.8	171.4
**LAM-FAS (Ours)**	**11.2**	**89.3**	**36.7**

## Data Availability

The data presented in this study are openly available in [CASIA-SURF] at [10.1109/CVPR.2019.00101], [CASIA-SURF CeFA] at [10.1109/WACV48630.2021.00122], [CASIA-FASD] at [10.1109/ICB.2012.6199754], reference number [3,40,41].
